# ADS-YOLO: A Multiscale Feature-Enhanced Network for Pepper Seedling Emergence Detection in Complex Plug-Tray Nursery Scenes

**DOI:** 10.3390/plants15213451

**Published:** 2026-11-05

**Authors:** Hua Yin, Mingyu Zeng, Ziwei Song, Yinglong Wang, Ziyi Liao, Yebao Wu, Li Li, Juan Liu, Ke Wang, Anping Xiong

**Affiliations:** 1 School of Software, Jiangxi Agricultural University, Nanchang 330045, China; 2 School of Computer and Information Engineering, Jiangxi Agricultural University, Nanchang 330045, China; 3 Ji’an Agricultural and Rural Development Service Center, Ji’an 343000, China; 4 Ji’an Institute of Agricultural Sciences, Ji’an 343000, China; 5 Qingyuan District Bureau of Agriculture and Rural Affairs, Ji’an 343000, China; 6 Ji’an Municipal Jinggangshan Agricultural Products Regional Public Brand Development Service Center, Ji’an 343000, China; 7 Ji’an Zerun Agricultural Development Co., Ltd., Ji’an 343000, China

**Keywords:** pepper seedlings, seedling emergence detection, tiny object detection, dynamic upsampling

## Abstract

Accurate detection of emerged seedlings is essential for intelligent management in the context of industrialized pepper seeding cultivation. Manual assessment of seedling emergence rates is inefficient and subjective. Meanwhile, dense seedling distributions and complex substrate backgrounds in plug trays often cause false positives and missed detections in vision-based systems. To address these issues, Adaptive-Dynamic-Spatial YOLO (ADS-YOLO), a compact detection model, was built based upon YOLO11n. First, an Adaptive Scale Encoding (ASE) module was introduced into the neck to preserve fine-grained spatial information through parallel variable-scale processing and fusion. Second, a Dynamic Scale Sequence Fusion (DYSF) module combined content-aware DySample upsampling with a scale-indexed three-dimensional feature representation to improve multiscale alignment and fusion. Third, a Spatial-Channel Position Attention (SCPA) module was used to emphasize seedling-related responses and suppress interference from substrate particles, water stains, and reflections. On the pepper seedling validation set obtained from a fixed 8:1:1 image-level data split, ADS-YOLO achieved a precision of 79.0%, a recall of 75.5%, and an mAP@0.5 of 78.1%, representing improvements of 3.3, 3.2, and 2.9 percentage points over YOLO11n. The model contained approximately 3.04 M parameters and required 8.1 GFLOPs. Furthermore, a prototype based on offline video-frame analysis was developed to display seedling detections and frame-level counts. The prototype achieved an offline processing throughput of 24.5 FPS and a per-frame end-to-end processing time of 40.8 ms. These measurement results indicate that the method proposed in this paper can be used in practical scenarios, and also lay the foundation for future real-time tracking and counting.

## 1. Introduction

Pepper is an economically important solanaceous crop used widely as food, medicine, and industrial material. Plug-tray nursery technology enables standardized, resource-efficient, and large-scale seedling production while ensuring quality, making it integral to pepper seedling cultivation [1]. The seedling emergence rate directly affects stand establishment, uniformity, and subsequent yield and serves as a key indicator for seed-quality evaluation and nursery management. Nevertheless, emergence assessment still relies heavily on manual observation, which is labor-intensive, time-consuming, subjective, and difficult to standardize [2,3,4,5]. This situation has seriously hindered the development of related industries.

Currently, with the advancement of computer technology, machine vision and deep learning have been increasingly applied to automated seedling monitoring in various scenarios [6,7,8,9]. For instance, Bai et al. developed a YOLOv5-based method for wheat seed germination detection [10], whereas Lang proposed an image-processing system for identifying foxtail millet germination and measuring radicle and plumule lengths [11]. Yao et al. improved YOLOv7 for tea-bud localization, pose estimation, and grading under variable illumination and occlusion [12]. Sun et al. proposed CSGD-YOLO for maize seed germination-status detection and reported an mAP@0.5 of 92.99% with 1.91 M parameters [13]. For pepper nursery applications, Shi et al. proposed Light-YOLO-Pepper to detect missing seedlings in plug trays, and Tasmara et al. developed a deep learning model for monitoring early germination stages in greenhouse-grown pepper seedlings [14,15]. These studies demonstrate the potential of compact YOLO-based networks for automated nursery monitoring.

Nevertheless, industrial plug-tray nursery environments remain challenging because newly emerged seedlings occupy only a small number of pixels, are densely distributed, may overlap, and often exhibit visual characteristics similar to soil, perlite, water stains, and reflective background regions. These factors can cause feature attenuation, multiscale misalignment, false positives, and missed detections, limiting the reliability of existing methods in practical production settings.

To mitigate the issues of feature attenuation and complex backgrounds in small object detection, recent advancements in the computer vision community have explored various multiscale feature fusion and attention mechanisms. For instance, dynamic alignment strategies and multiscale feature extraction networks have been proposed to preserve high-resolution spatial details that are often lost during downsampling, thereby improving the detection of small targets [16,17]. Furthermore, incorporating attention-based fusion has proven effective in bridging the semantic gap and suppressing background clutter for tiny objects, particularly in real-world agricultural scenarios [18]. Additionally, unified methods optimized for densely distributed small objects have demonstrated strong capabilities in enhancing localization in complex environments without significantly increasing computational costs [19]. Despite these generalized advancements, existing multiscale fusion and attention techniques often struggle with the extreme visual similarities between seedlings and perlite/soil substrates, as well as the dense distribution specific to industrial pepper plug-tray nurseries. This leaves a critical research gap for a customized, compact detection architecture tailored to address these compounded agricultural scenarios.

Compared to relevant pepper plug-tray methods, this study addresses a distinct research gap. For target definition, we explicitly focus on newly emerged seedlings with fully unfolded cotyledons rather than missing seedlings or seedlings at earlier germination stages. Regarding the dataset setting, our dataset captures compounded real-world challenges (e.g., reflective perlite and uneven illumination) instead of controlled uniform backgrounds. Finally, our evaluation protocol extends beyond standard metrics by incorporating a fixed 8:1:1 image-level split, zero-shot cross-crop generalization tests, and offline video-frame analysis, providing a baseline for potential agricultural deployment. To address these challenges, this study developed an Adaptive-Dynamic-Spatial YOLO (ADS-YOLO) based on YOLO11n for pepper seedling emergence detection. The main contributions of this study are summarized as follows: (1) A real-world pepper seedling dataset was constructed under complex industrial plug-tray nursery scenarios to reflect practical production challenges. (2) A compact detection model, Adaptive-Dynamic-Spatial YOLO (ADS-YOLO), was developed based on YOLO11n. This model integrates three core modules: an Adaptive Scale Encoding (ASE) module for parallel multiscale processing, a Dynamic Scale Sequence Fusion (DYSF) module for content-aware alignment, and a Spatial-Channel Position Attention (SCPA) module to suppress complex substrate interference. (3) The proposed model was systematically evaluated through comprehensive comparative, ablation, visualization, and cross-crop experiments, and was successfully integrated into a prototype emergence detection and counting interface to demonstrate its practical effectiveness.

## 2. Results

### 2.1. Experimental Environment

Model training and testing were conducted in the same computational environment to improve reproducibility. The software and hardware configuration is listed in Table 1.

The principal training settings are presented in Table 2. During image preprocessing, the original high-resolution images (2560 × 1440 pixels) were processed using a target input size of 640 × 640 pixels to balance computational efficiency and deployment feasibility. Aspect-ratio-preserving resizing and padding were applied as required by the Ultralytics preprocessing pipeline. During evaluation, predicted bounding-box coordinates were mapped back to the original image dimensions by accounting for the resizing scale and padding offsets. The same preprocessing and coordinate-restoration procedures were applied to all evaluated models. To improve robustness to variations in object scale and spatial context, Mosaic data augmentation was applied during training. To ensure stable optimization for the tiny target detection task and to improve experimental reproducibility, the model was initialized with official YOLO11n pretrained weights (trained on the COCO dataset) and trained using the customized network structure defined by the YAML file. The optimizer option was set to ‘auto’, which selected AdamW and automatically determined the effective initial learning rate and optimizer momentum under the Ultralytics training framework. A linear learning-rate decay schedule with three warm-up epochs was used. The random seed was set to 0 for the representative training run, while the additional ADS-YOLO runs used different random seeds, as described in Section 2.3. Furthermore, Mosaic data augmentation was disabled during the final 10 epochs (close_mosaic = 10) to facilitate stable fine-tuning and enhance final localization convergence. The remaining settings followed the YOLO11n default configuration unless otherwise specified.

### 2.2. Evaluation Metrics

Model performance was evaluated using precision (*P*), recall (*R*), and mean average precision at an IoU threshold of 0.5 (mAP@0.5). Because the dataset contains one target class, mAP is numerically equivalent to AP for that class.

Precision (*P*) is the proportion of true positives (*TP*) among all detections predicted as pepper seedlings and is calculated as follows:

*P* = *TP*/(*TP* + *FP*) (1)

Recall (*R*) is the proportion of actual pepper seedlings that are correctly detected and is calculated as follows:

*R* = *TP*/(*TP* + *FN*) (2)

In Equations (1) and (2), TP denotes the number of correctly detected seedlings, *FP* denotes the number of background regions incorrectly detected as seedlings, and *FN* denotes the number of actual seedlings that remain undetected.

**Figure 1:**
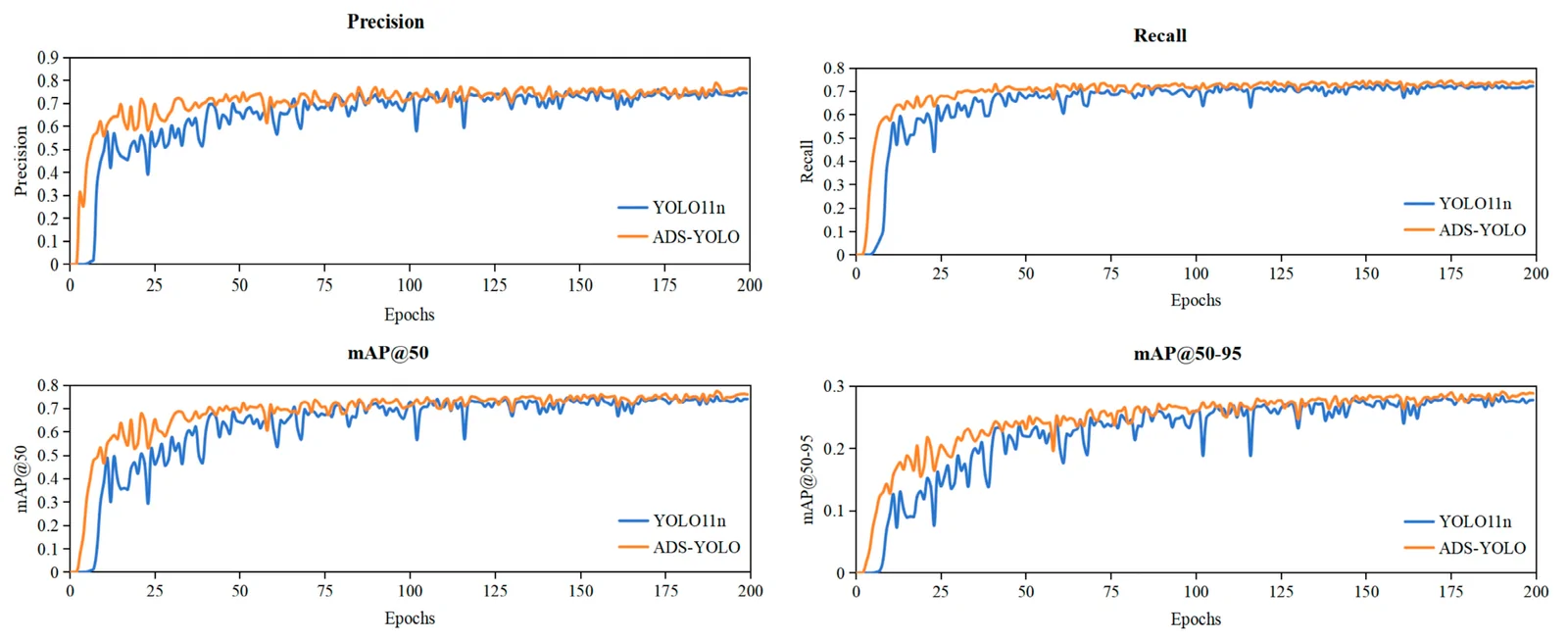
Validation-set precision, recall, mAP@0.5, and mAP@0.5:0.95 curves of YOLO11n and ADS-YOLO during training. The curves correspond to a single YOLO11n run and the ADS-YOLO run with the highest validation mAP@0.5 among three independent runs. Both models used the same fixed dataset split.

To ensure a consistent evaluation procedure, the confidence threshold used to report precision and recall for each model, including the baseline and ablation variants, was determined by maximizing the F1 score on the validation set. For instance, this optimal confidence threshold was 0.159 for our proposed ADS-YOLO and dynamically varied between 0.124 and 0.159 for the ablation variants. A unified Non-Maximum Suppression (NMS) IoU threshold of 0.7 was consistently applied across all evaluations. For each training run, the best.pt checkpoint was selected according to the highest validation fitness score, as defined by the Ultralytics framework. Among the three independent ADS-YOLO runs, the run with the highest validation mAP@0.5 was selected for the convergence-curve comparison presented in Figure 1.

Average precision (AP) summarizes the precision-recall curve across confidence thresholds. In this study, mAP at an IoU threshold of 0.5 (mAP@0.5) was adopted as the primary accuracy metric. AP and mAP are calculated using Equations (3) and (4), respectively:

(3) $$\mathrm{AP}=\int_{0}^{1}P(R)\mathrm{dR}$$

(4) $$\mathrm{mAP}=\frac{1}{N}\sum_{i=1}^{N}AP_{i}$$

In Equation (4), N denotes the number of classes. Since this study considers only one target class (N = 1), mAP is numerically equal to AP at the same IoU threshold. Here, P(R) denotes precision as a function of recall. In addition, mAP@0.5:0.95 was calculated by averaging AP over ten IoU thresholds ranging from 0.50 to 0.95 in increments of 0.05, providing a more stringent assessment of bounding-box localization accuracy.

### 2.3. Model Performance

Among the three independent training runs of ADS-YOLO, the run with the highest validation mAP@0.5 achieved a precision of 79.0%, a recall of 75.5%, and an mAP@0.5 of 78.1% on the validation set. As shown in Figure 1, the selected ADS-YOLO run generally exhibited higher validation metric values than the YOLO11n run during training. Both models used the same fixed split into training, validation, and test sets. The curves in Figure 1 correspond to the selected ADS-YOLO run and the single YOLO11n run.

To evaluate the run-to-run stability of the proposed method, ADS-YOLO was independently trained three times using different random seeds while maintaining the same fixed dataset split. Across these runs, the validation-set results, expressed as mean ± standard deviation, were 78.9% ± 0.4% for precision, 75.1% ± 0.4% for recall, 77.9% ± 0.3% for mAP@0.5, and 30.0% ± 0.2% for mAP@0.5:0.95. In comparison, the YOLO11n baseline was trained once using the same fixed dataset split. The relatively small standard deviations indicate limited run-to-run variability in ADS-YOLO under the evaluated conditions. However, these standard deviations characterize only the run-to-run variability of ADS-YOLO and do not establish a statistically significant performance difference between ADS-YOLO and the YOLO11n baseline.

**Figure 2:**
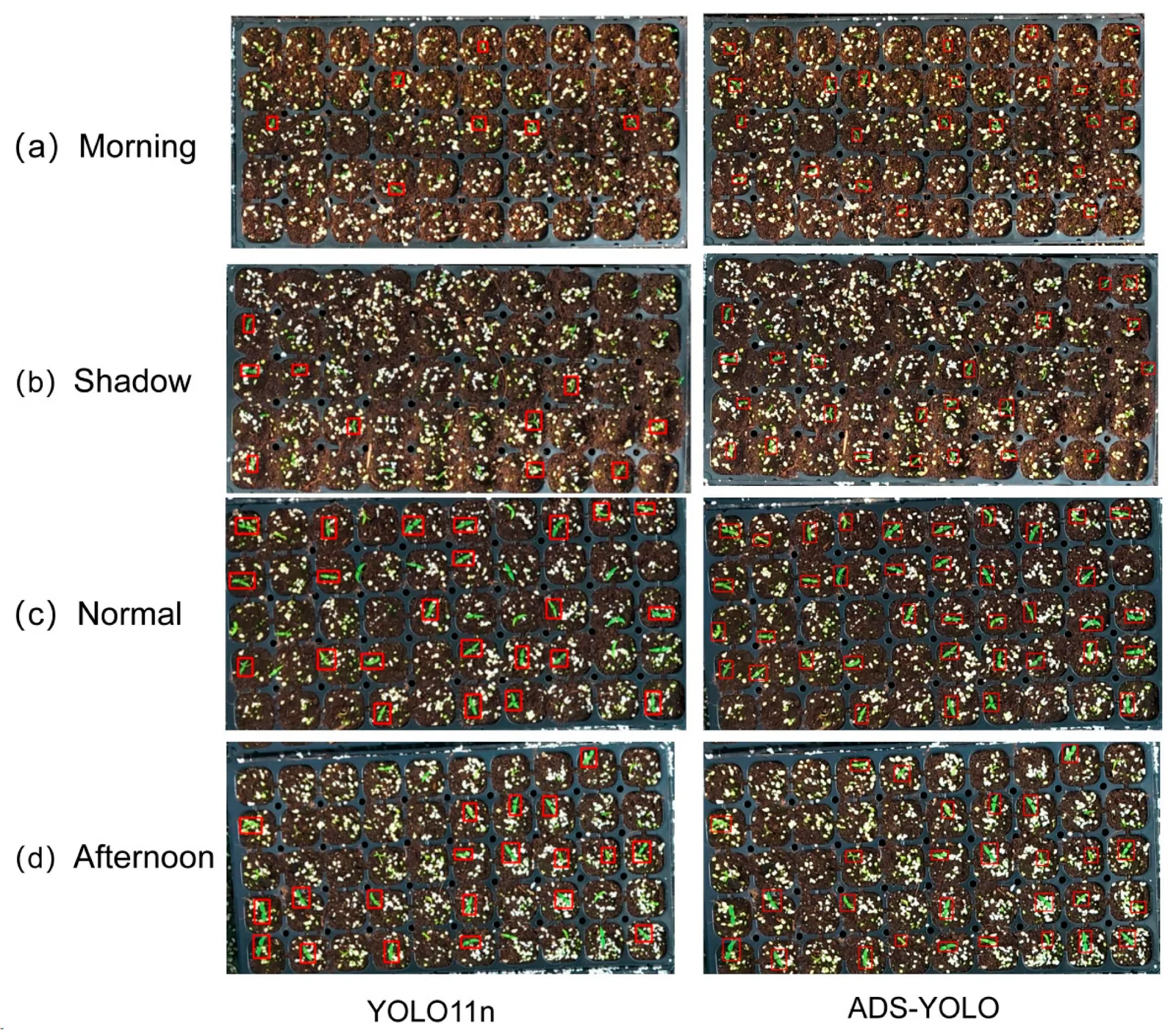
Qualitative detection comparison between the YOLO11n baseline (left) and the proposed ADS-YOLO (right) under different environmental and illumination conditions.

To examine whether the quantitative improvements translated into practical performance, four representative greenhouse conditions were selected for qualitative evaluation, as shown in Figure 2: (a) low illumination in the morning, (b) shadows and structural occlusion, (c) approximately uniform illumination, and (d) strong directional illumination in the afternoon.

Across these diverse illumination and environmental scenarios, ADS-YOLO retained stable detections in the presence of low contrast, reflective perlite, and uneven tray shadows. The bounding boxes generally localized newly emerged seedlings closely, with fewer visible false positives and missed detections than the baseline. These observations support the robustness of ADS-YOLO under the evaluated conditions, although deployment-level validation additionally requires continuous counting and trajectory tracking error analysis under dynamic operational conditions.

### 2.4. Ablation Experiments

Ablation experiments were conducted on the pepper seedling validation set to quantify the independent contribution of ASE, DYSF, and SCPA and the performance of their joint integration. YOLO11n was used as the baseline, and all reported variants were trained using the same parameter settings. The results are presented in Table 3.

The YOLO11n baseline achieved a precision of 75.7%, a recall of 72.3%, and an mAP@0.5 of 75.2%. Adding ASE increased recall to 74.9% (+2.6 percentage points) and mAP@0.5 to 76.4%, consistent with improved retention of tiny-seedling details. Adding DYSF produced the largest precision gain, increasing precision to 78.5% (+2.8 percentage points), which is consistent with improved spatial alignment and reduced localization ambiguity. Adding SCPA alone increased precision and recall to 76.9% and 72.9%, respectively, but yielded an mAP@0.5 of 75.1%, which was 0.1 percentage points below the baseline. One possible explanation for this slight decrease is that applying spatial-channel attention in isolation may amplify irrelevant background features, although this mechanism has not been verified through a dedicated error analysis. Thus, the benefit of SCPA appears more evident when it is integrated with the other modules than when it is used independently. Furthermore, inference latency and model file size are reported alongside accuracy, parameter count, and FLOPs in Table 3 to characterize the computational characteristics of the evaluated variants.

The complete ADS-YOLO model achieved the highest precision (79.0%), recall (75.5%), and mAP@0.5 (78.1%) among the evaluated ablation variants. These results indicate complementary, but not uniformly additive, effects among the modules. Compared with YOLO11n, ADS-YOLO increased the parameter count from 2.58 M to 3.04 M (+17.7%) and the computational complexity from 6.3 to 8.1 GFLOPs (+28.6%). The accuracy gain should therefore be interpreted together with the additional computational cost and verified through latency measurements on the intended deployment hardware.

### 2.5. Comparison with Other Models

ADS-YOLO was compared with YOLOv8n, YOLOv9t, YOLOv10n, YOLO11n, YOLO12n, and YOLO13n under the same experimental setting. To ensure experimental reproducibility and a fair evaluation, all baseline models (including the recent YOLO12n and YOLO13n) were sourced directly from the official Ultralytics framework (version 8.3.189). Their implementations strictly followed the official default network structures and hyperparameter configurations without any architectural modifications. The results are reported in Table 4.

Among the evaluated models, ADS-YOLO achieved the highest precision (79.0%), recall (75.5%), and mAP@0.5 (78.1%). Its mAP@0.5 exceeded those of the YOLO11n baseline, YOLO12n, and YOLO13n by 2.9, 3.3, and 3.9 percentage points. However, ADS-YOLO has 3,038,694 parameters and an inference latency of 28.6 ms, compared with 2,582,347 parameters and 9.94 ms for the YOLO11n baseline. Therefore, although ADS-YOLO maintains a relatively compact architecture, its improved detection accuracy comes at the cost of a higher parameter count and longer inference latency than YOLO11n.

The comparison suggests that feature processing tailored to tiny, densely distributed seedlings can be advantageous over generic architectures under the tested plug-tray nursery conditions. However, this conclusion is restricted to the present dataset and experimental protocol.

### 2.6. Visual Comparison

Representative samples containing dense seedlings, sparse seedlings, and different illumination conditions were selected for qualitative comparison among representative object-detection models, with particular emphasis on YOLO11n and ADS-YOLO.

#### 2.6.1. Detection Results of Different Models

**Figure 3:**
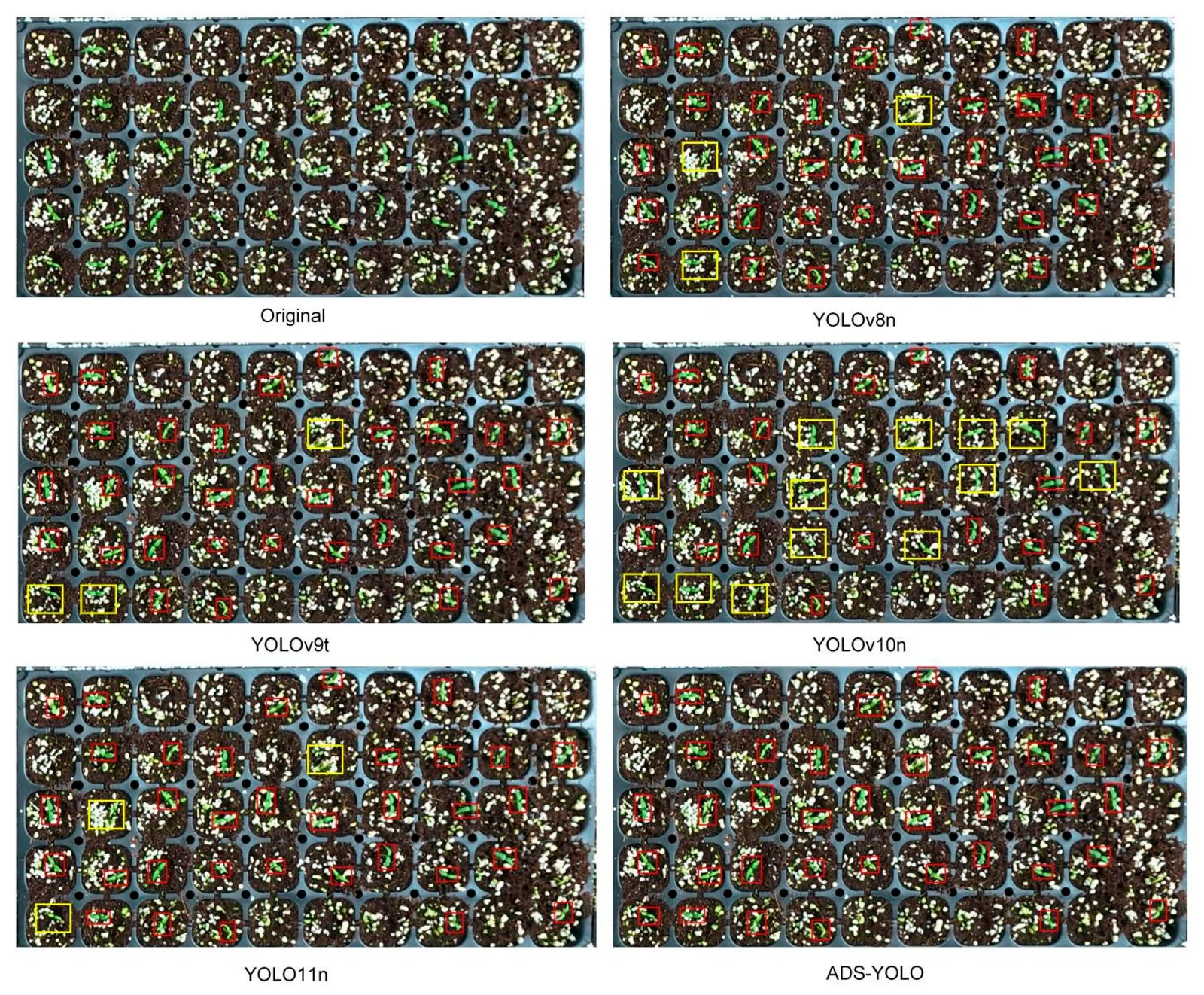
Visual comparison of multiple object-detection models under challenging nursery conditions. The red boxes indicate standard detection results, while the yellow boxes highlight regions with missed detections or false positives.

Figure 3 compares the qualitative detection results of multiple object-detection models under challenging nursery conditions. In densely populated and partially occluded regions, several baseline models produced visible missed detections or false positives and had difficulty separating adjacent seedlings, whereas ADS-YOLO more consistently distinguished overlapping instances, consistent with the fine-grained feature preservation of ASE. Under uneven illumination, ADS-YOLO also showed fewer visible errors on reflective substrate particles. These qualitative results indicate improved stability under the evaluated non-ideal conditions.

#### 2.6.2. Grad-CAM Heatmap Analysis

**Figure 4:**
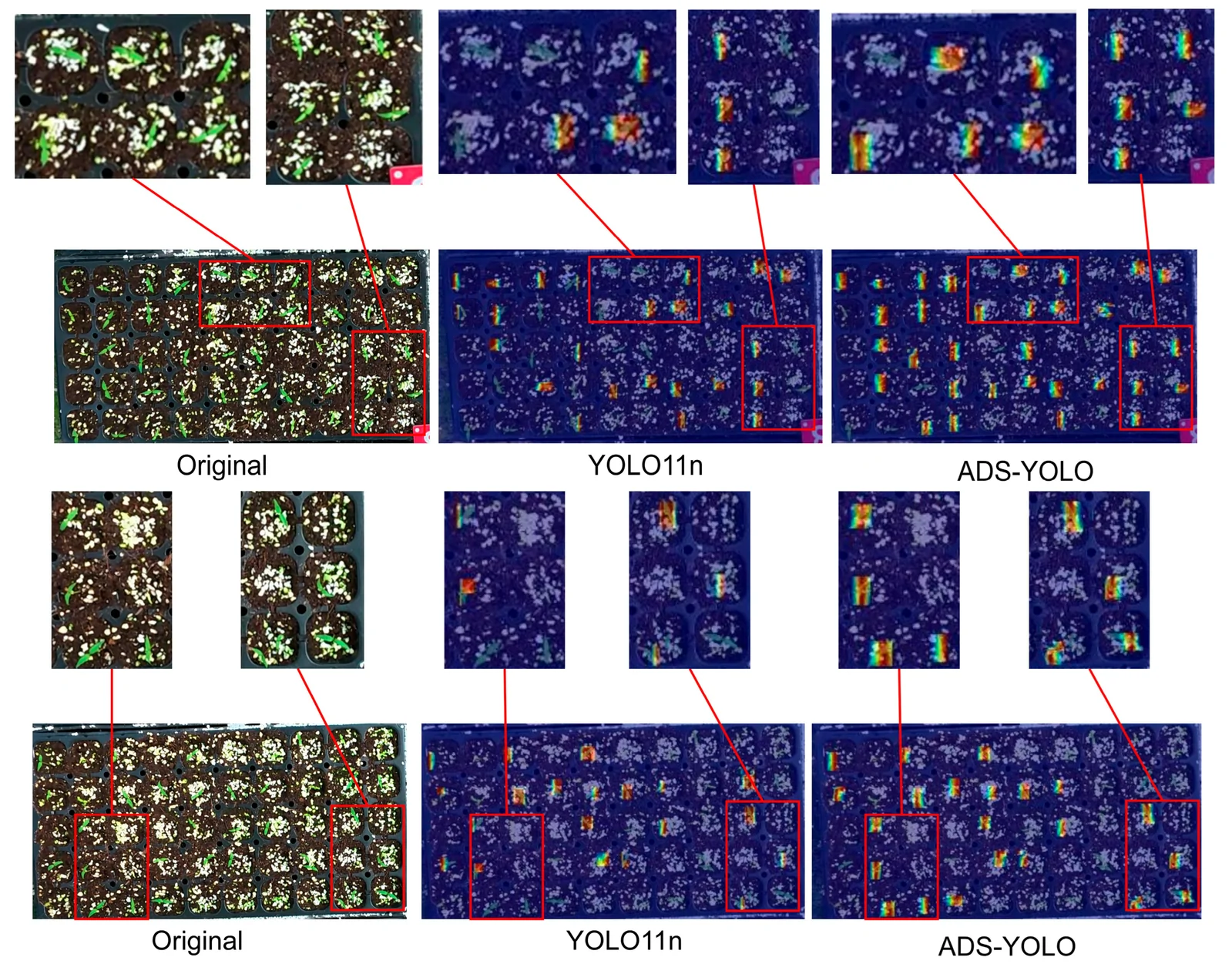
Grad-CAM heatmap comparison between YOLO11n and ADS-YOLO.

Grad-CAM was used to visualize class-related activation patterns in the detection network, as shown in Figure 4. Warm colors indicate regions with stronger model responses, whereas cool colors indicate weaker responses. For dense tiny seedlings, YOLO11n exhibited weaker and more fragmented activation patterns, with several local responses shifted away from the target centers. ADS-YOLO produced more concentrated activations around individual seedlings and weaker responses in reflective substrate regions. These heatmaps provide qualitative support for improved target focusing and background suppression, although they should not be interpreted as independent quantitative evidence of detection accuracy.

### 2.7. Cross-Crop Applicability Experiments

To examine whether the proposed architecture is applicable beyond pepper seedlings, ADS-YOLO and YOLO11n were evaluated on two additional plug-tray crop datasets, namely spinach and muskmelon. The spinach and muskmelon images were collected at the Zerun Nursery Base in Ji’an, Jiangxi Province, China, following the same greenhouse image-acquisition protocol used for the pepper seedling dataset, including the camera setup and sampling conditions described in Section 4.1. Similar to the pepper seedling dataset, these images were collected from the emergence stage to the third day after germination. These crops differ from pepper in leaf shape, canopy size, planting density, and occlusion patterns, providing complementary test cases for cross-crop applicability. To assess cross-crop transferability, the proposed ADS-YOLO and baseline models were directly evaluated on the spinach and muskmelon datasets using the weights trained on the pepper seedling dataset, without any retraining or fine-tuning. For precision, recall, and F1-score evaluation, the confidence threshold determined for each model on the pepper validation set was retained unchanged for the spinach and muskmelon datasets, without any target-crop-specific adjustment. The input resolution of 640 × 640 and the NMS IoU threshold of 0.7 were also kept consistent with the pepper evaluation. Specifically, 50 images of spinach and 100 images of muskmelon were utilized entirely as the test sets. Since no training was involved in this phase, a traditional dataset split ratio was not applicable.

As shown in Table 5, ADS-YOLO improved all reported metrics on the muskmelon dataset, achieving an mAP@0.5 of 96.5%, 1.7 percentage points above YOLO11n. On the more densely distributed spinach dataset, ADS-YOLO achieved an mAP@0.5 of 73.2% and a recall of 74.2%, representing improvements of 1.6 and 3.4 percentage points, respectively, over the baseline.

Precision on the spinach dataset decreased from 71.2% to 69.5%, whereas recall and F1 increased to 74.2% and 71.8%, respectively. This trade-off indicates that the improved model recovered additional overlapping seedlings at the cost of more false positives. A detailed false-positive analysis revealed that these errors are closely related to the extremely small size of the newly emerged spinach seedlings, which are noticeably smaller than the pepper seedlings used for training. Because the targets are so microscopic, the model occasionally misclassified similar-sized background noise as seedlings. Specifically, the highly reflective perlite particles scattered across the dark substrate and tiny green algae patches were the main background elements mistakenly detected, leading to the slight decrease in precision. Overall, these results provide preliminary cross-crop evidence for ADS-YOLO under the evaluated conditions; further validation on larger and more diverse datasets is needed to establish its broader applicability.

### 2.8. Prototype System for Pepper Seedling Detection and Counting

**Figure 5:**
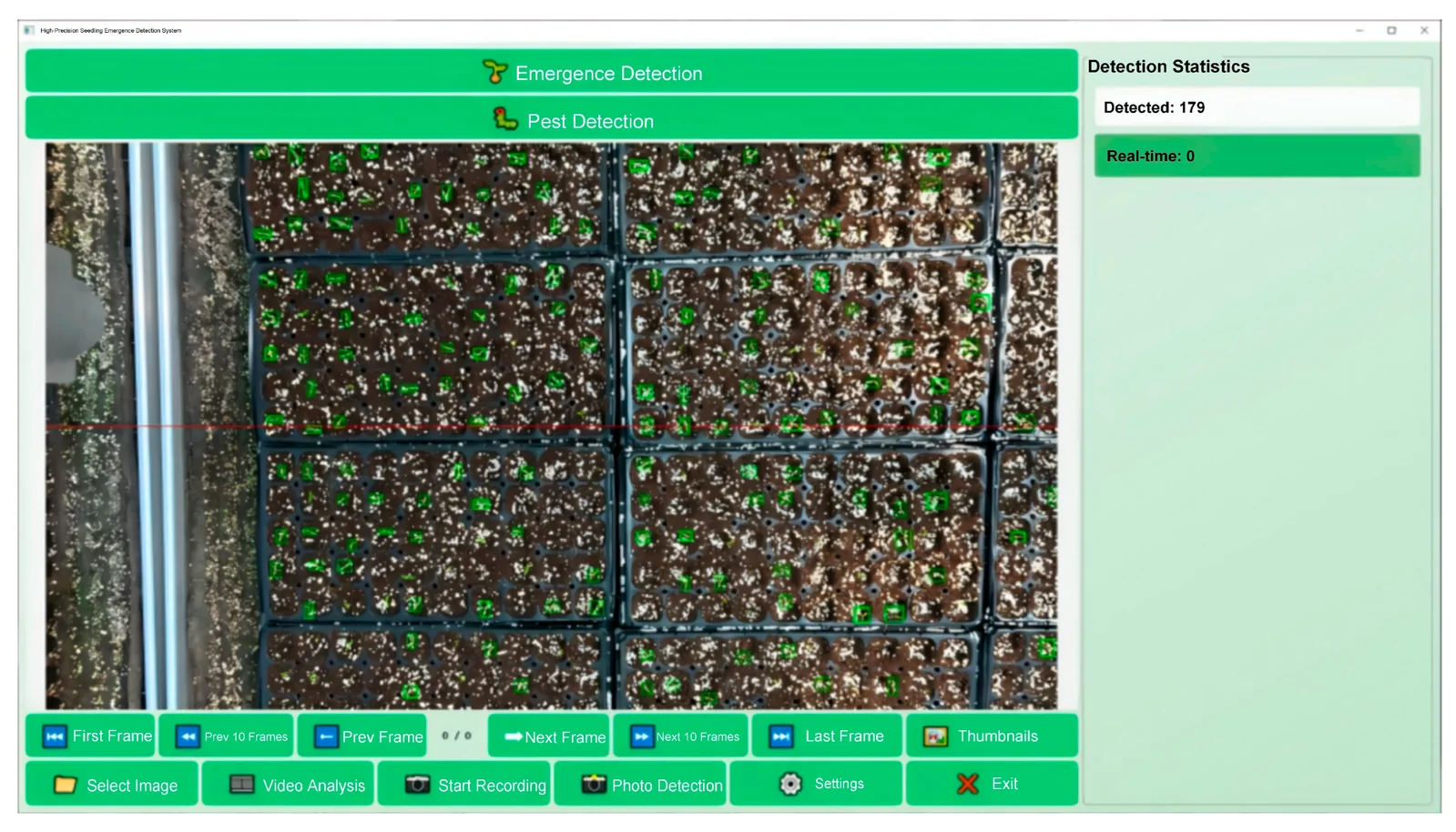
User interface of the prototype system for offline pepper seedling detection and frame-level count visualization. Note: The system interface language shown is English, with modules designed for seedling emergence detection, count display, and video analysis. Unrelated functional tabs (e.g., pest detection) belong to an extended monitoring suite and are not evaluated in this study.

A prototype pepper seedling detection and counting system was developed to support the practical deployment of ADS-YOLO, as shown in Figure 5. The interface comprises an image display area, a statistical summary area, and a functional control area. The central panel visualizes the detection results with green bounding boxes around identified seedlings; the statistical panel displays the number of seedlings detected in the current frame; and the lower controls allow users to import image or video streams and export detection logs.

To evaluate the practical utility of the prototype in mobile nursery environments, video sequences captured in the greenhouse were processed on a mobile tablet using a frame-extraction-based offline analysis approach rather than online multi-object tracking. Under the tested tablet hardware and software configuration, the prototype achieved an offline processing throughput of 24.5 FPS, corresponding to an average end-to-end processing time of 40.8 ms per frame. The prototype application used approximately 850 MB of system RAM during offline video-frame analysis. This system-level memory measurement is distinct from the model-level metric reported in Table 3. These results demonstrate the feasibility of offline frame-level seedling detection and counting on the tested device, rather than validated real-time tracking or cross-frame unique-seedling counting. The graphical user interface thus supports non-contact seedling detection and frame-level count visualization for nursery management.

## 3. Discussion

### 3.1. Interpretation of Model Performance

**Figure 6:**
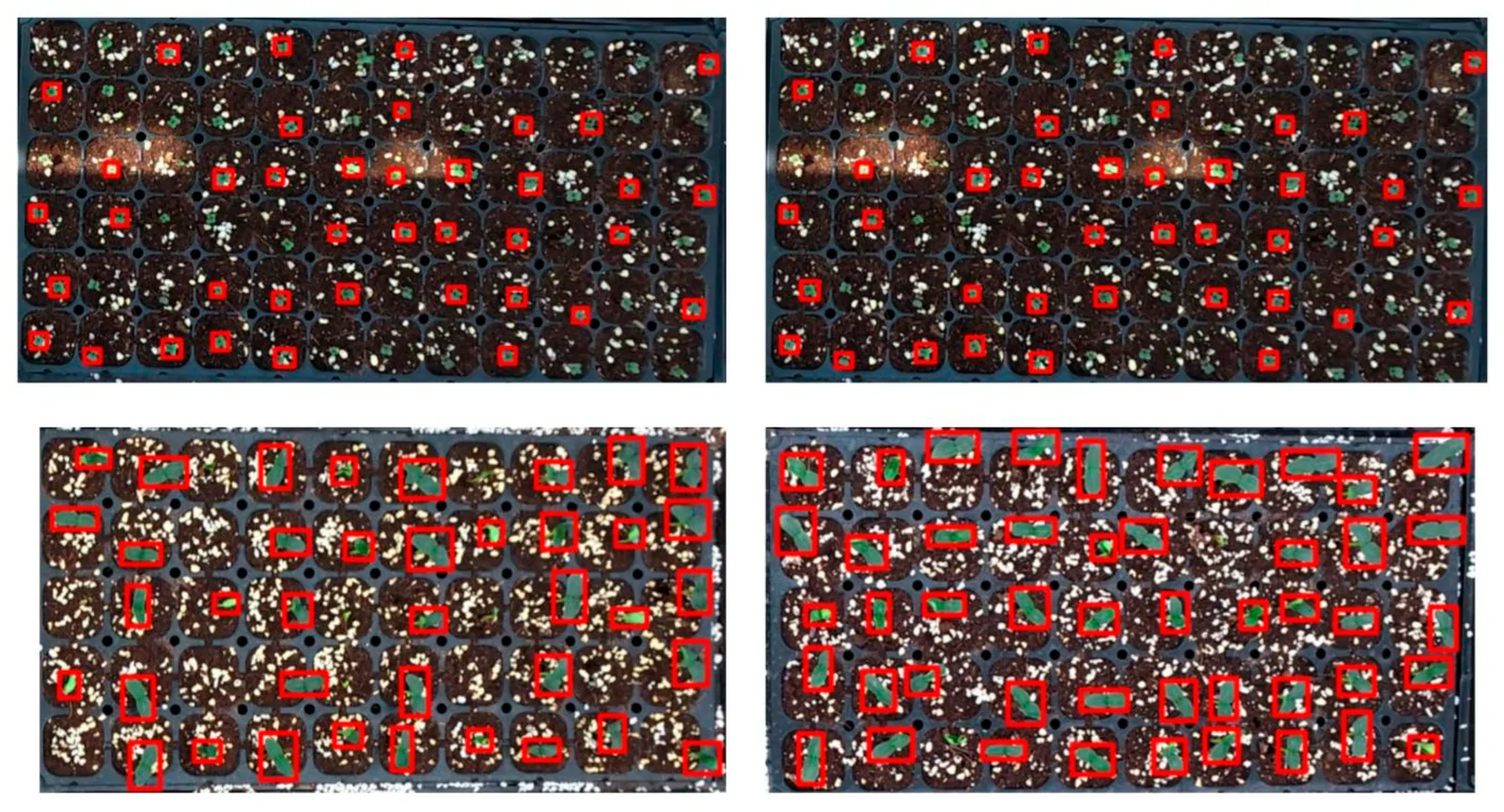
Detection results of the proposed ADS-YOLO model on different plug-tray crops ((**top**) spinach, (**bottom**) muskmelon).

The spinach and muskmelon experiments, as visualized in Figure 6, indicate that the proposed architecture can remain effective for plug-tray crops with different canopy structures and planting densities. Notably, the model exhibited superior detection performance on muskmelon seedlings compared to spinach. This performance disparity is primarily driven by the distinct morphological characteristics of the two crops. Muskmelon seedlings feature larger, prominent cotyledons and leaves with strong structural boundaries that contrast sharply against the soil background, making their features easier for the network to extract and localize. Conversely, spinach seedlings are considerably smaller with fine-grained leaf structures that are more susceptible to background interference, such as perlite particles. The diminished visual footprint and weaker morphological features of the spinach seedlings inherently increase the difficulty of accurate bounding box regression and classification, resulting in relatively lower detection accuracy.

### 3.2. Limitations and Future Work

**Figure 7:**
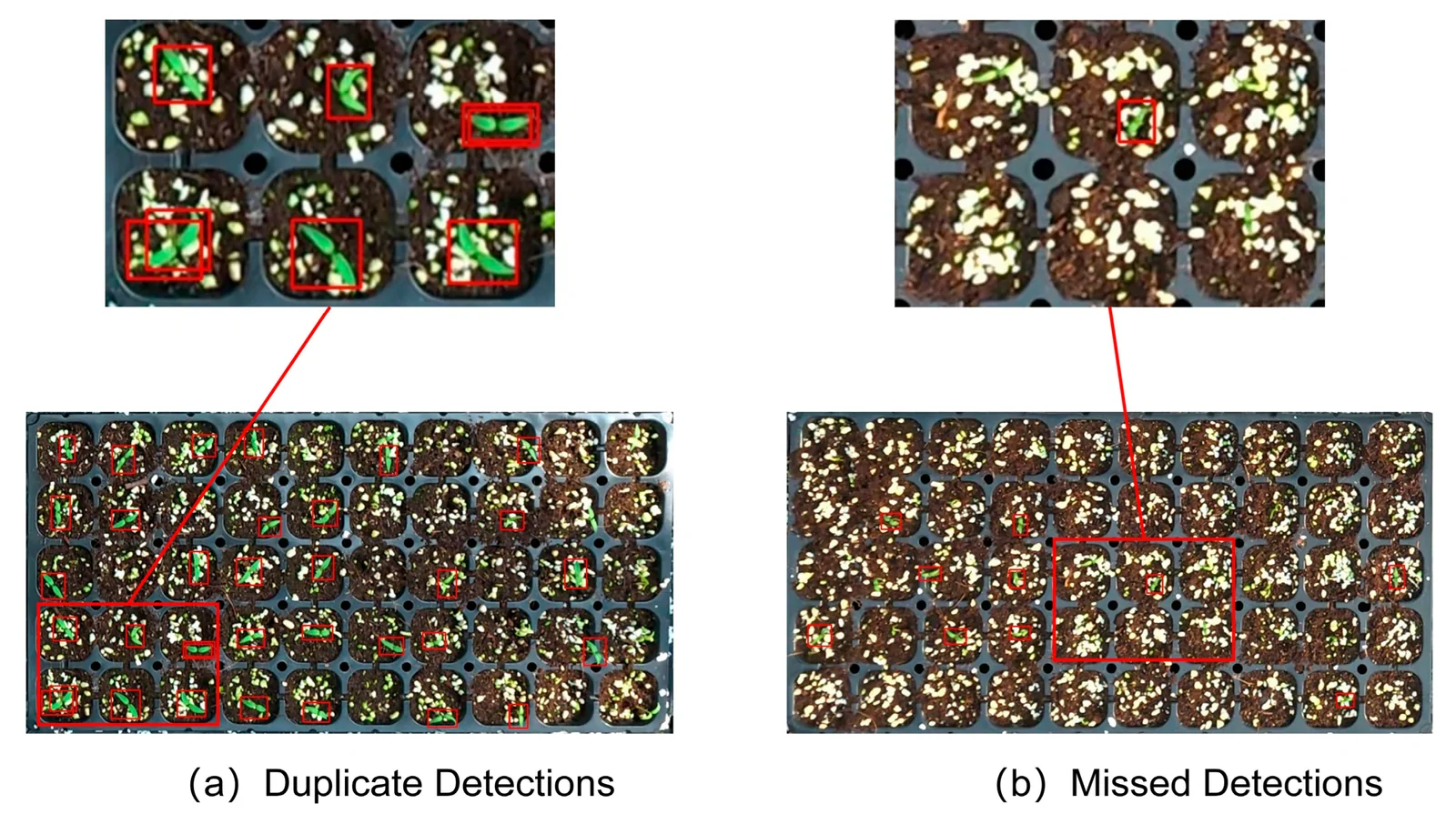
Representative failure cases of ADS-YOLO under challenging background conditions.

ADS-YOLO still exhibits characteristic failure modes under extreme conditions, as shown in Figure 7. When the cotyledons of a single seedling are spatially separated or appear visually fragmented, a local leaf region may be detected as an independent seedling, resulting in duplicate detections. Conversely, an extremely small newly emerged seedling adjacent to highly reflective white perlite may be missed because its weak edge and color cues are overwhelmed by the background.

Despite the promising detection results, the current study has limitations regarding the data acquisition methodology. Specifically, the system relies on mechanically timed static photographic captures rather than continuous video monitoring. This discrete sampling approach can lead to statistical inaccuracies when assessing the entire seedbed. Without continuous video tracking, variations in the mechanical movement or timing can easily result in duplicate counting of the same pepper seedlings across overlapping frames or missed detections in unsampled gaps. Future work should consider integrating continuous video streams with multi-object tracking algorithms to ensure more reliable and accurate whole-seedbed statistics.

The relatively large difference between mAP@0.5 and mAP@0.5:0.95 may be partly attributed to the sensitivity of tiny-object detection to bounding-box localization errors. For newly emerged pepper seedlings, even small deviations in the predicted bounding-box boundaries can substantially reduce the intersection over union (IoU), particularly at stricter IoU thresholds. In addition, dense seedling distributions and complex substrate backgrounds may further increase the difficulty of precise localization. Consequently, the lower mAP@0.5:0.95 is consistent with the challenges of achieving high-precision localization for tiny seedlings, even when satisfactory detection performance is obtained at IoU = 0.5. However, the reported metrics alone do not establish the exact contribution of each potential source of localization error.

## 4. Materials and Methods

### 4.1. Pepper Seedling Image Acquisition and Dataset Construction

Seedlings of two major pepper cultivars grown in Jiangxi Province, namely ‘Hangjiao Zaocui’ and ‘Yicihong’, were selected as the experimental subjects. Images were acquired in late July and early August 2025 in a greenhouse at the Zerun Nursery Base in Ji’an, Jiangxi Province, using a DJI Action 5 camera (DJI, Shenzhen, China) with a resolution of 2560 × 1440 pixels. During acquisition, the camera was positioned vertically 50 cm above the plug trays and mounted on an automated sprinkler boom traveling at a constant speed of approximately 0.5 m/s. To reduce redundancy between adjacent video frames, frames were extracted from the continuous video recordings at 4-s intervals. Given the approximate camera travel speed of 0.5 m/s, this interval corresponds to an estimated displacement of 2.0 m between consecutive sampled frames.

**Figure 8:**
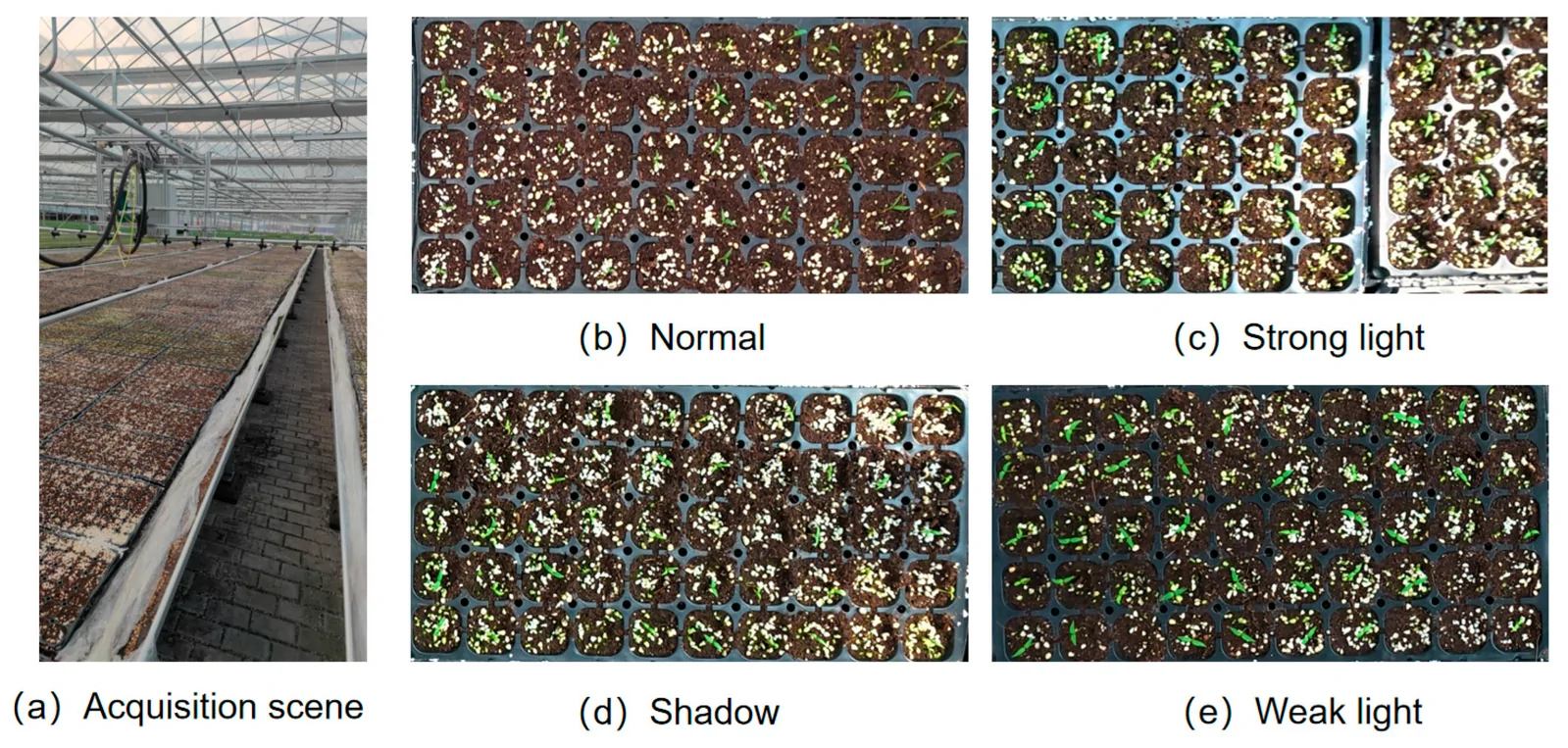
Pepper seedling image-acquisition environment and representative images under different illumination conditions.

The image acquisition spanned three consecutive days, commencing upon the appearance of the first seedling and concluding on the third day of growth. To ensure a robust evaluation, the final dataset comprised 730 images, including 454 images of ‘Hangjiao Zaocui’ and 276 images of ‘Yicihong’. By capturing images during the morning, at noon, and in the afternoon, the dataset comprehensively covers four representative illumination conditions: normal, strong light, shadow, and weak light (as illustrated in Figure 8).

During dataset construction, a seedling was visually defined as “emerged” and manually annotated with the class label “SEED” only when its two cotyledons were fully unfolded. To guarantee high annotation quality and consistency, the bounding boxes were independently drawn by two agricultural researchers and subsequently cross-verified and standardized by a third inspector. Finally, the annotation files were generated in YOLO format, and the dataset was divided into training, validation, and test sets at a ratio of 8:1:1. Although the estimated displacement may reduce overlap between adjacent frames along a single continuous traversal, it does not guarantee non-overlapping fields of view or spatial independence across different acquisition days or repeated traversals of the same nursery beds. Consequently, overlapping frames and repeated observations of the same seedlings may remain in the dataset, including across the random image-level splits, as discussed in Section 3.2. The validation set was used to monitor the training process and select the best model weights, whereas the test set was excluded from model selection and hyperparameter tuning. Specifically, the 8:1:1 split ratio corresponds to 584 images for training, 73 for validation, and 73 for testing.

Based on a preliminary count of the annotation files, the dataset contained approximately 77,600 annotated seedling instances, including about 63,200 instances in the training set, 7900 in the validation set, and 6600 in the test set. The approximate counts were rounded independently. Regarding the object-size distribution, a preliminary analysis of the training-set annotations indicated that all assessed bounding boxes (100%) had areas smaller than 1024 square pixels, equivalent to the area of a 32 × 32-pixel square, after aspect-ratio-preserving resizing to a 640 × 640 input resolution. These preliminary statistics highlight the challenges of accurately detecting and localizing newly emerged seedlings in complex plug-tray nursery environments. Furthermore, images were acquired from multiple plug-tray segments through the continuous spatial sampling procedure described above, without assuming uniform spatial coverage across individual trays.

### 4.2. ADS-YOLO Model

**Figure 9:**
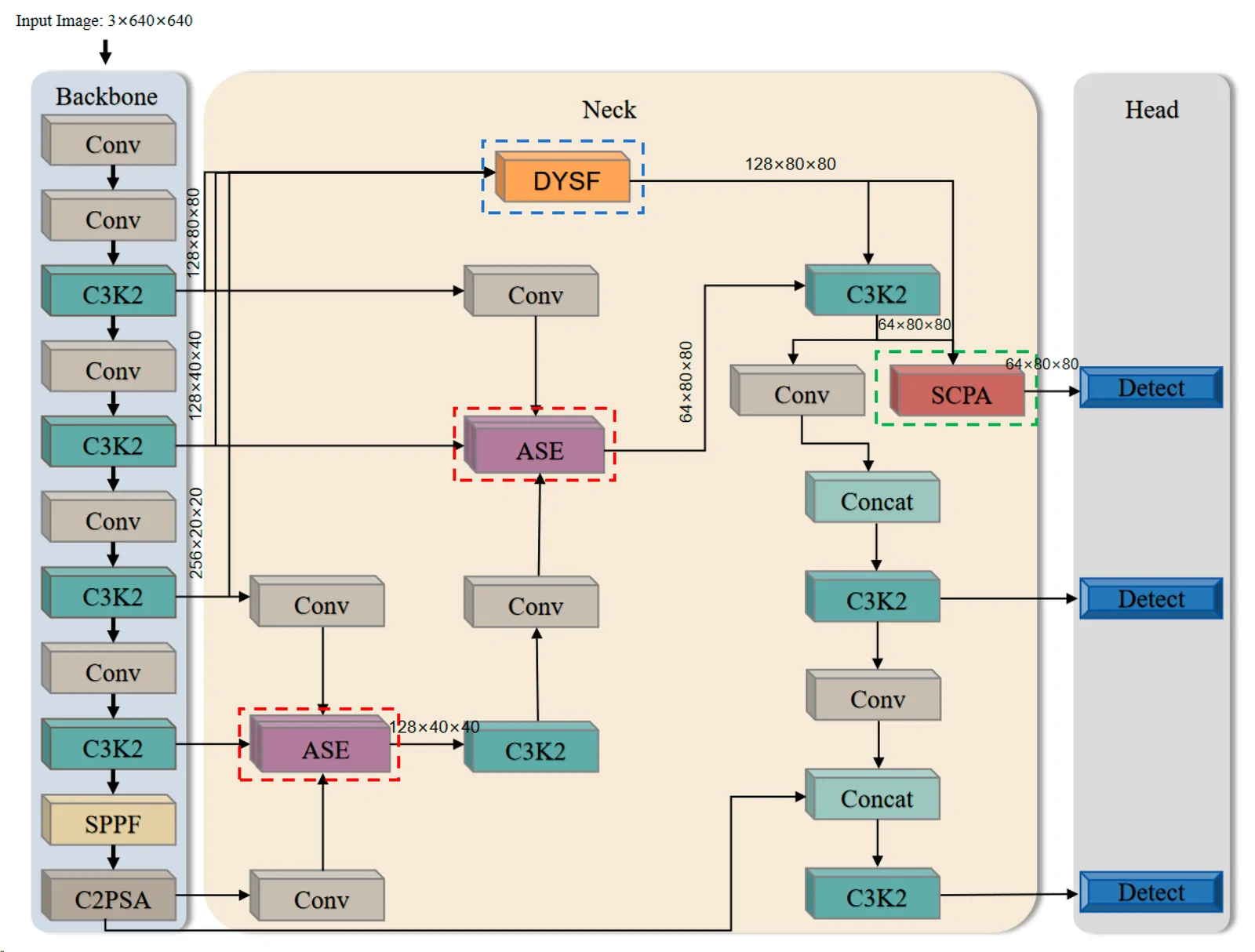
Network architecture of the ADS-YOLO pepper seedling emergence detection model. Note: The solid arrows indicate the direction of data flow, and blocks with different colors represent distinct computational operations. The dashed boxes highlight the three proposed modules: the red dashed boxes denote the Adaptive Scale Encoding (ASE) modules, the blue dashed box indicates the Dynamic Scale Sequence Fusion (DYSF) module, and the green dashed box marks the Spatial-Channel Position Attention (SCPA) module.

YOLO11n was adopted as the baseline network for constructing ADS-YOLO, a pepper seedling emergence detection model designed for plug-tray nursery environments. First, the ASE module uses a multibranch parallel structure to combine fine-grained local texture information with higher-level semantic information. Second, the DYSF module aligns the P3, P4, and P5 feature maps to a common spatial resolution using DySample, stacks them as a scale-indexed pseudo-three-dimensional tensor, and performs channel transformation followed by scale-wise aggregation. Finally, the SCPA module sequentially recalibrates channel and positional responses to emphasize seedling-related features and suppress background interference. The overall network architecture is illustrated in Figure 9. Specifically, the ASE module is positioned early in the neck to preserve and encode fine-grained spatial information immediately after backbone extraction. The DYSF module is strategically embedded within the fusion pathways to provide content-aware dynamic alignment precisely where resolution transitions (upsampling) occur. Finally, the SCPA module is placed exclusively before the high-resolution P3 detection head because P3 is structurally responsible for detecting microscopic targets; this targeted placement maximizes the suppression of complex substrate noise while strictly controlling the overall computational overhead (GFLOPs), avoiding unnecessary latency at the P4 and P5 heads. At the layer level, the original YOLO11n top-down feature fusion stages, involving nearest-neighbor upsampling and concatenation at layers 11–12 and 14–15, were reorganized using convolutional projections and two ASE modules at layers 13 and 17 of ADS-YOLO. For an input resolution of 640 × 640, the first ASE module receives feature maps of 128 × 80 × 80, 128 × 40 × 40, and 128 × 20 × 20 (C × H × W), producing an output of 384 × 40 × 40. The second ASE module receives feature maps of 64 × 160 × 160, 128 × 80 × 80, and 64 × 40 × 40, producing an output of 256 × 80 × 80. The DYSF module at layer 25 receives P3, P4, and P5 features of 128 × 80 × 80, 128 × 40 × 40, and 256 × 20 × 20, respectively, and generates a fused feature map of 128 × 80 × 80. The SCPA module at layer 26 receives two feature maps of 64 × 80 × 80 and 128 × 80 × 80, respectively, and produces a 64 × 80 × 80 feature map for the P3 detection branch. Regarding initialization, the ASE modules introduce no learnable parameters, whereas the trainable convolutional and normalization layers in DYSF and SCPA use the initialization procedures provided by their respective PyTorch and Ultralytics implementations. The DySample operators follow the initialization procedure defined in their implementation. No additional experiment-specific weight initialization was applied. The parameter counts in Table 3 were calculated separately for each ablation configuration. For the complete ADS-YOLO model, the same network configuration was used for the parameter counts reported in Table 3 and Table 4, yielding 3,038,694 parameters, with a computational complexity of 8.1 GFLOPs.

#### 4.2.1. Adaptive Scale Encoding Module (ASE)

**Figure 10:**
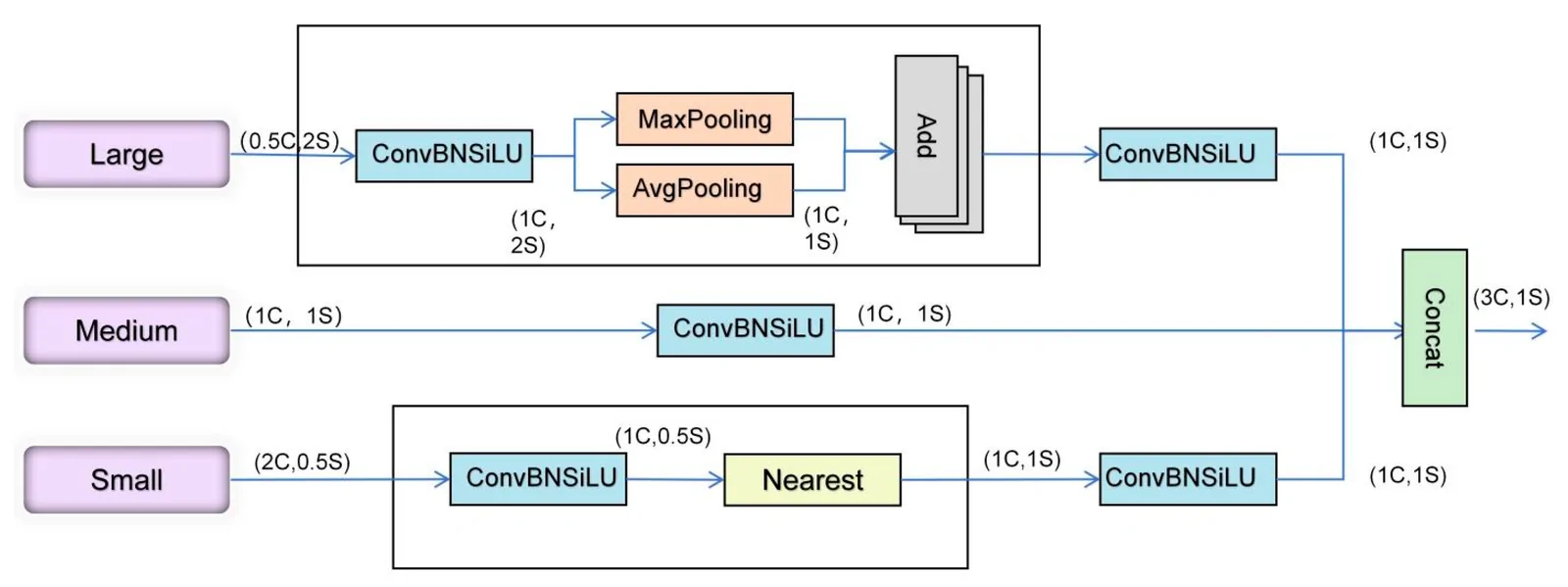
Architecture of the Adaptive Scale Encoding (ASE) module.

The architecture of the ASE module is shown in Figure 10. The three aligned branches are fused by channel-wise concatenation to provide a detail-rich multiscale representation for the subsequent detection layers.

To improve recognition of tiny objects in densely distributed and overlapping scenes, the ASE module adopts a parallel multiscale enhancement strategy to mitigate the attenuation of tiny-object information in deep networks. Relative to the FPN/PAN-style neck used in the YOLO11n baseline, which performs sequential top-down and bottom-up feature fusion through operations including upsampling and concatenation, ASE adopts a parallel alignment strategy for three input feature maps. Specifically, the three features are spatially aligned through pooling and nearest-neighbor interpolation and then concatenated along the channel dimension. This description concerns the structural difference between ASE and the specific YOLO11n implementation; it does not imply that FPN/PAN architectures are limited to a single fusion strategy or demonstrate the general superiority of ASE over other FPN/PAN variants. No strictly parameter-matched experiment comparing ASE with alternative FPN/PAN variants was conducted in this study. The core operations are defined as follows:

First, the input feature maps are projected to a common channel dimension and aligned to a common spatial resolution. For the large-scale feature map *F_L_*, max pooling and average pooling are combined to retain salient local responses and global contextual information during downsampling. For the medium-scale feature map *F_M_*, a convolutional layer directly standardizes the channel dimension. For the small-scale feature map *F_S_*, nearest-neighbor interpolation is used for upsampling because it avoids the smoothing introduced by bilinear interpolation and better preserves the local edges of tiny objects. The alignment operations for the three branches are formulated as follows:

*F*′*_L_* = *φ_L_*[Pool_max_(*F_L_*,*τ*) + Pool_avg_(*F_L_*,*τ*)] (5)

*F*′*_M_* = *φ_M_*(*F_M_*) (6)

*F*′*_S_* = *φ_S_*[Interp_nearest_(*F_S_*,*τ*)] (7)

Here, *τ* denotes the scale-alignment factor used to standardize the spatial resolutions of the three branches. Specifically, as depicted in Figure 9, to align both the large-scale branch (2S) and the small-scale branch (0.5S) with the medium-scale baseline (1S), τ is uniformly set to 2 for both the pooling stride and the interpolation scale factor; *φ_L_*, *φ_M_*, and *φ_S_* denote the 1 × 1 convolution operations applied to the large-, medium-, and small-scale branches, respectively; and *F*′*_L_*, *F*′*_M_*, and *F*′*_S_* denote the aligned features after channel unification. The three outputs have identical spatial resolutions, providing the basis for subsequent concatenation.

Finally, the three aligned features are concatenated along the channel dimension to generate a multiscale feature representation enriched with local detail and contextual information:

*F*_ASE_ = Concat(*F*′*_L_*,*F*′*_M_*,*F*′*_S_*) (8)

Here, *F*_ASE_ denotes the output of the ASE module, while *F*′*_L_*, *F*′*_M_,* and *F*′*_S_* denote the processed large-, medium-, and small-scale feature maps, respectively. Specifically, as each of the three aligned branches (*F*′*_L_*, *F*′*_M_*, and *F*′*_S_*) is standardized to a channel dimension of *C*, the concatenated multiscale feature *F*_ASE_ results in an exact output channel dimension of 3*C*. *F*_ASE_ retains the spatial resolution of the aligned branches and integrates complementary information from different receptive fields: the large-scale branch preserves geometric details, the medium-scale branch supplements structural information, and the small-scale branch provides broader semantic context. This complementary representation improves the detection of tiny seedlings in dense and overlapping scenes.

#### 4.2.2. Dynamic Scale Sequence Fusion Module (DYSF)

**Figure 11:**
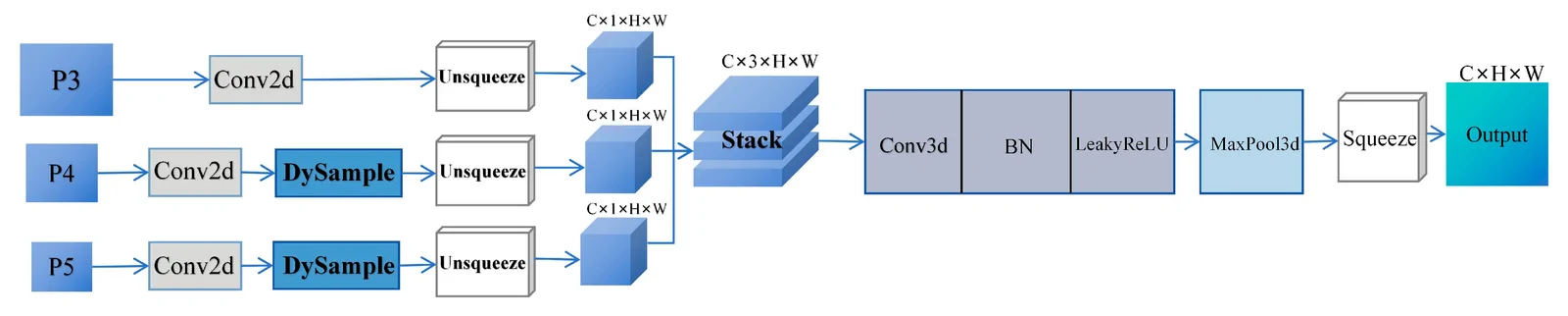
Architecture of the Dynamic Scale Sequence Fusion (DYSF) module.

The DYSF module was designed to reduce feature misalignment, semantic mixing, and insufficient scale-wise interaction during multiscale fusion in the FPN. It performs three successive operations: content-aware spatial alignment, scale-indexed feature transformation, and scale-wise aggregation. Its architecture is shown in Figure 11.

To reduce feature blurring caused by fixed-interpolation upsampling, the lightweight DySample operator [21] replaces conventional nearest-neighbor or bilinear interpolation. DySample predicts sampling offsets from the input features and therefore provides content-aware spatial alignment while retaining fine-grained details of tiny seedlings.

First, the input feature maps *P*_3_, *P*_4_, and *P*_5_ are projected to a common channel dimension and aligned to the spatial resolution of *P*_3_:

*F*_3_ = *φ*_1×1_(*P*_3_),  *F*_4_ = *D*_↑2_[*φ*_1×1_(*P*_4_)],  *F*_5_ = *D*_↑4_[*φ*_1×1_(*P*_5_)] (9)

Here, *φ*_1×1_(·) denotes a 1 × 1 two-dimensional convolution that projects features at different scales to a common channel dimension, and C.D_↑s_(·) denotes DySample upsampling with scale factor s, where s = 2 for P_4_ and s = 4 for P_5_. The aligned outputs are denoted by F_3_, F_4_, and F_5_.

The aligned two-dimensional feature maps are then stacked along a newly introduced depth dimension to construct a scale-indexed pseudo-three-dimensional tensor:

*T* = Stack(*F*_3_, *F*_4_, *F*_5_) (10)

Here, Stack(·) denotes stacking along the depth dimension, producing a 5D tensor *T* ∈ ℝ*^B^*^×*C*×*D*×*H*×*W*^, where *B* is the batch size, *C* is the channel dimension, *H* × *W* is the spatial resolution, and the depth *D* = 3 explicitly corresponds to the three aligned feature scales (P3, P4, and P5).

The stacked tensor is subsequently processed by a 1 × 1 × 1 three-dimensional convolution, batch normalization, and LeakyReLU activation. Because the convolution kernel spans one element along the depth dimension, this operation performs channel transformation at each scale position while preserving the scale-indexed structure. Cross-scale selection is performed in the subsequent depth-wise max-pooling operation. The transformation is formulated as follows:

*T*′ = LeakyReLU_0.1_(ℬ[*Φ*_3*D*_(*T*)]) (11)

Here, *Φ*_3D_(·) denotes a three-dimensional convolutional layer with a kernel size of (1,1,1); B(·) denotes three-dimensional batch normalization, which stabilizes optimization. LeakyReLU with a negative slope of 0.1 is used as the activation function to introduce nonlinearity.

A 3D max-pooling operation is subsequently applied to compress the depth dimension, retain the strongest scale response, and reduce computational redundancy:

*Y* = *S*[MaxPool3D(*T*′)] (12)

where MaxPool3D(·) denotes three-dimensional max pooling with a kernel size of (3,1,1). Because the preceding 1 × 1 × 1 convolution independently transforms channels at each scale without mixing them along the depth dimension, this subsequent MaxPool3D operation is essential for producing cross-scale interaction. By applying a (3,1,1) kernel, it acts as a competitive cross-scale selection mechanism, computing the maximum activation along the depth dimension (*D* = 3) for each spatial location and channel. This explicitly routes the most salient features from either P3, P4, or P5 into the final fused representation. S(·) denotes the squeeze operation used to remove the singleton depth dimension, yielding the fused two-dimensional feature map Y ∈ ℝ^C×H×W^.

Furthermore, Section 2.4 presents an ablation study comparing the YOLO11n baseline with a variant incorporating the complete DYSF module, which combines DySample-based spatial alignment, scale-indexed feature processing, and scale-wise aggregation. This comparison evaluates the effect of the integrated DYSF module rather than isolating the contributions of its individual components.

#### 4.2.3. Spatial-Channel Position Attention Module (SCPA)

To enhance semantic consistency and suppress interference from complex backgrounds, including substrate particles, water stains, and reflections, an SCPA module was introduced.

**Figure 12:**
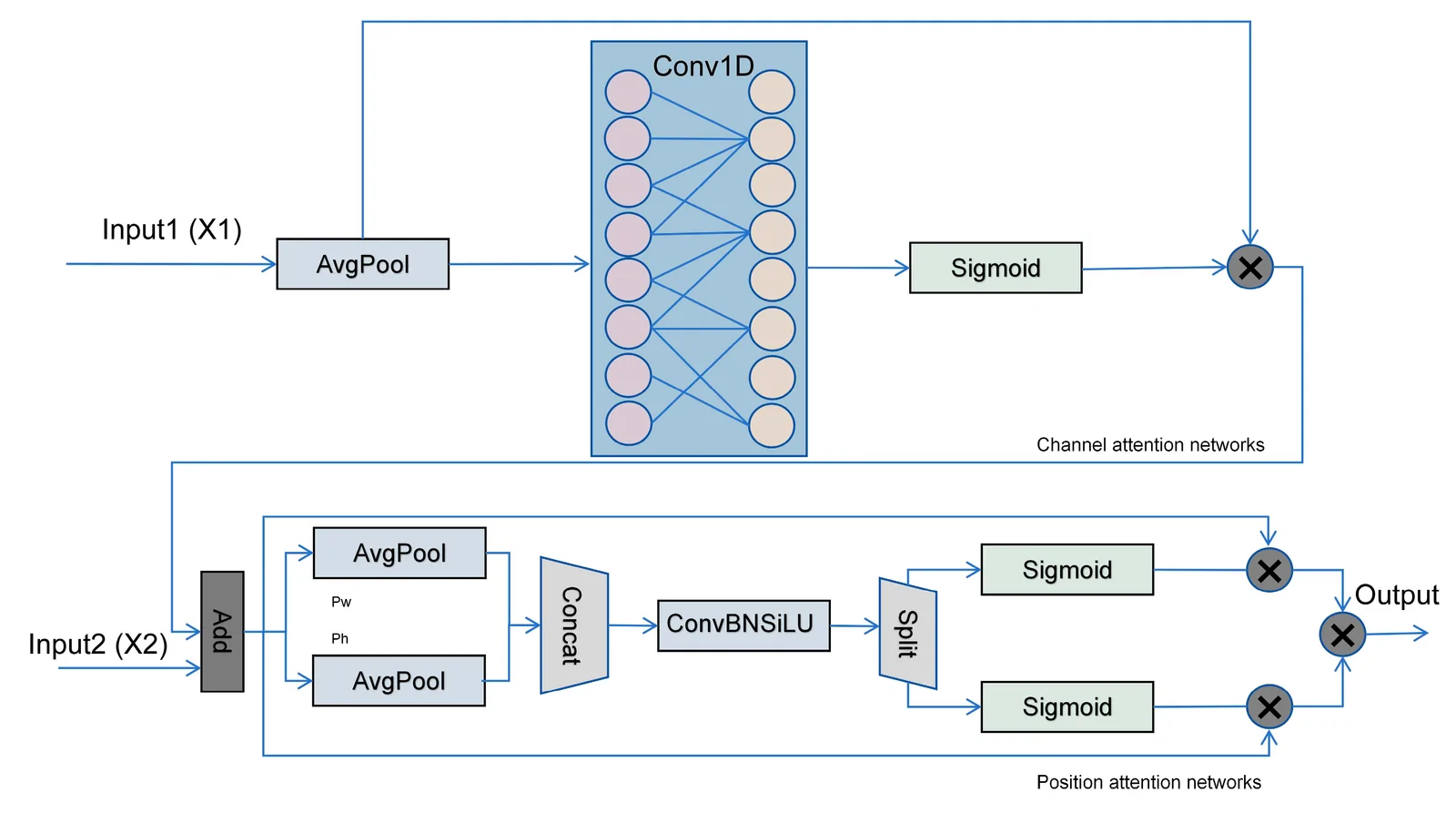
Architecture of the Spatial-Channel Position Attention (SCPA) module.

The module adopts a cascaded architecture derived from Efficient Channel Attention (ECA) [22] and Coordinate Attention (CA) [23] that recalibrates features along the channel and spatial dimensions, as illustrated in Figure 12.

The channel-attention submodule captures inter-channel dependencies using global average pooling without channel reduction and a one-dimensional convolution, thereby estimating channel weights while retaining complete channel information.

*k* = *Ψ*(*C*) = |(log_2_*C* + *b*)/*γ*|_odd_ (13)

Here, *C* denotes the number of input channels, and *b* and *γ* are hyperparameters, typically set to *b* = 1 and *γ* = 2. The operator | ⋅ |_odd_ rounds the computed value to the nearest odd integer, ensuring a symmetric odd-sized convolution kernel.

Based on the adaptive kernel size, channel-attention weights are generated and applied to the input feature map as follows:

*F*_ca_ = *X*_1_⊗*σ*{Conv1D*_k_*[AvgPool(*X*_1_)]} (14)

Here, *X*_1_ denotes the input feature map from the P3 neck branch, and *X*_2_ (introduced in the subsequent position-attention submodule) denotes the fused feature map from the DYSF module; AvgPool(·) denotes global average pooling; Conv1D_k_(·) denotes a one-dimensional convolution with kernel size k for modeling local inter-channel dependencies; σ(·) denotes the Sigmoid function; and ⊗ denotes element-wise channel weighting. The resulting channel-enhanced feature map is denoted by F_ca_.

First, an intermediate feature tensor *E* ∈ ℝ*^C^*^×*H*×*W*^ is computed by adding the channel-enhanced feature *F_ca_* and the DYSF module output *X*_2_ (i.e., *E* = *F_ca_* + *X*_2_). Then, global average pooling is applied to *E* along the two spatial directions to produce the positional encoding vectors *p_h_* and *p_w_*. For a given channel *c*, the pooling operations along the vertical (height) and horizontal (width) directions are formulated as follows:

(15) $$p_{h}(h)=\frac{1}{W}\sum_{0\leq w<W}E_{c}(h,w),\;p_{w}(w)=\frac{1}{H}\sum_{0\leq h<H}E_{c}(h,w)$$

Here, *H* and *W* denote the spatial height and width of the feature map. The boundary indexing variables *h* ∈ [0, *H* − 1] and *w* ∈ [0, *W* − 1] represent the vertical and horizontal spatial coordinates. To align the dimensions for feature aggregation, the vertical descriptor *p_h_* ∈ ℝ*^C^*^×*H*×1^ is spatially transposed to $p_{h}^{T}\in R^{C\times 1\times H}$. Subsequently, $p_{h}^{T}$ and *p_w_* ∈ ℝ*^C^*^×1×*W*^ are concatenated along the spatial width dimension. The combined tensor is then processed by a 1 × 1 convolution, batch normalization (ℬ), and a ReLU activation function (δ) to generate the intermediate spatial attention map *a* ∈ ℝ*^C^*^/*r*×1×(*H*+*W*)^:

(16) $$a=\delta (B(\mathrm{Conv}2D([p_{h}^{T},p_{w}])))$$

where [⋅,⋅] denotes concatenation along the spatial width axis, and *r* is the channel reduction ratio.

Finally, tensor *a* is split along the spatial width dimension into two independent descriptors *s_h_*′ and *s_w_*′. The vertical descriptor is transposed back to its original orientation (*s_h_′^T^*). Both descriptors are then processed by independent 1 × 1 convolutions (*F_h_* and *F_w_*) to restore the channel dimension and pass through Sigmoid mapping (*σ*) to yield the final attention maps *s_h_* ∈ ℝ*^C^*^×*H*×1^ and *s_w_* ∈ ℝ*^C^*^×1×*W*^. These spatial attention maps are applied to the intermediate feature, yielding the final SCPA output *F_SCPA_*:

*s_h_*′, *s_w_*′ = *Split*(*a*, axis = width) (17)

*s_h_* = *σ*(*F_h_*(*s_h_′^T^*)),  *s_w_* = *σ*(*F_w_*(*s_w_*′)) (18)

*F_SCPA_* = (*F_ca_* + *X*_2_)⊗*s_h_*⊗*s_w_* (19)

Through channel recalibration and orthogonal positional weighting, SCPA strengthens target-related responses while reducing sensitivity to redundant background features.

## 5. Conclusions

This study developed ADS-YOLO for pepper seedling emergence detection in industrial plug-tray nursery environments characterized by tiny targets, dense distributions, and strong background interference. The ASE, DYSF, and SCPA modules were introduced to improve fine-grained feature preservation, multiscale alignment and aggregation, and target-focused feature weighting, respectively. On the pepper validation set, ADS-YOLO achieved a precision of 79.0%, a recall of 75.5%, and an mAP@0.5 of 78.1%, corresponding to improvements of 3.3, 3.2, and 2.9 percentage points over YOLO11n. A tablet-based prototype was also evaluated using offline video-frame analysis, achieving a processing throughput of 24.5 FPS and an average end-to-end processing time of 40.8 ms per frame under the tested conditions. These results characterize offline frame-level processing rather than validated real-time tracking or cross-frame unique-seedling counting.

The complete model achieved the highest precision and mAP@0.5 among the evaluated ablation variants and showed favorable performance on spinach and muskmelon datasets, indicating its preliminary potential for cross-crop generalization. Nevertheless, to definitively confirm its broad applicability to multiple plug-tray crops, large-scale independent hold-out tests across different production sites, dates, and cultivars are required. Future work will expand the dataset to encompass these broader spatiotemporal conditions and investigate model pruning, quantization, and further optimization for deployment on resource-constrained edge devices.

## Figures and Tables

**Table 1:** Software and hardware configuration of the experimental environment.

Environment Configuration	Parameter
Operating system	Windows 10
CPU	Intel Xeon E5-1620 v3 (Intel Corporation, Santa Clara, CA, USA)
GPU	NVIDIA GeForce RTX 3060 (12 GB) (NVIDIA Corporation, Santa Clara, CA, USA)
Programming language	Python 3.8.2
Deep learning framework	PyTorch 2.3; Ultralytics 8.3.189 [20]
Operating platform	CUDA 12.7

**Table 2:** Hyperparameter settings for model training.

Settings	Parameters	Values
Training settings	Batch size	16
	Epochs	200
	Optimizer	Auto (AdamW)
Hyperparameters	Initial learning rate	0.01
	Weight decay	0.0005
	Momentum	0.937
	Warm-up epochs	3.0
	Mixed-precision training	True
	Random seed	0
	Close mosaic (epochs)	10
	Mosaic	1.0
	Mixup	0.0
	Input resolution	640 × 640
	Pretrained initialization	YOLO11n (COCO)
	Learning-rate schedule	Linear

**Table 3:** Ablation results for the proposed modules.

Model	ASE	DYSF	SCPA	P (%)	R (%)	mAP@0.5 (%)	mAP@0.5:0.95 (%)	Parameters	FLOPs (G)	Latency (ms)	Model File Size (MB)
YOLO11n (baseline)	×	×	×	75.7	72.3	75.2	28.4	2,582,347	6.3	9.94	5.2
YOLO11+ASE	√	×	×	76.7	74.9	76.4	28.6	2,733,403	7.2	19.9	5.5
YOLO11+DYSF	×	√	×	78.5	73.0	76.0	28.8	2,629,995	6.6	11.2	5.3
YOLO11+SCPA	×	×	√	76.9	72.9	75.1	28.1	2,599,574	6.3	16.7	5.3
YOLO11+ASE +DYSF	√	√	×	77.5	74.3	76.5	29.1	2,998,683	7.6	22.0	6.1
YOLO11+ASE +SCPA	√	×	√	78.5	75.4	77.8	29.8	2,960,070	7.4	10.5	6.0
YOLO11+DYSF +SCPA	×	√	√	77.9	74.5	76.8	29.6	2,622,518	6.5	17.4	5.3
ADS-YOLO (ours)	√	√	√	**79.0**	**75.5**	**78.1**	**30.1**	3,038,694	8.1	28.6	6.1

Note: Model file size refers to the storage size of the saved model checkpoint in megabytes (MB), rather than GPU memory consumption or runtime RAM usage. Bold values indicate the best results.

**Table 4:** Performance comparison with representative object-detection models.

Model	P (%)	R (%)	mAP@0.5 (%)	mAP@0.5:0.95 (%)	Parameters	Latency (ms)
YOLOv8n	76.2	72.3	74.5	27.5	2,684,563	9.06
YOLOv9t	75.8	73.3	75.0	28.4	1,730,019	28.5
YOLOv10n	72.6	71.6	74.7	27.5	2,265,363	6.24
YOLO11n	75.7	72.3	75.2	28.4	2,582,347	9.94
YOLO12n	76.4	72.6	74.8	28.4	2,508,539	18.3
YOLO13n	75.2	73.0	74.2	28.1	2,448,090	26.9
ADS-YOLO	**79.0**	**75.5**	**78.1**	**30.1**	3,038,694	28.6

Note: Bold values indicate the best results.

**Table 5:** Cross-crop applicability results.

Model	P (%)	R (%)	F1 (%)	mAP@0.5 (%)	mAP@0.5:0.95 (%)	Size (MB)
YOLO11n-Spinach	71.2	70.8	71.0	71.6	31.3	5.2
ADS-YOLO-Spinach	69.5	74.2	71.8	73.2	33.0	6.1
YOLO11n-Muskmelon	92.5	93.3	92.9	94.8	60.1	5.2
ADS-YOLO-Muskmelon	94.5	94.9	94.7	96.5	70.2	6.1

## Data Availability

The datasets generated and analyzed during this study are not publicly available because they are still being expanded and curated.

## References

[1] Kong D., Liu Y., Cui W., Wang Y., Zhang X. (2025). A lightweight method for detecting sowing quality in pepper plug-tray nurseries based on an improved YOLOv8n. Trans. Chin. Soc. Agric. Mach..

[2] Genze N., Bharti R., Grieb M., Schultheiss S.J., Grimm D.G. (2020). Accurate machine learning-based germination detection, prediction and quality assessment of three grain crops. Plant Methods.

[3] Ligterink W., Hilhorst H.W.M., Kleine-Vehn J., Sauer M. (2017). High-throughput scoring of seed germination. Plant Hormones: Methods and Protocols.

[4] Malik A., Ram B., Arumugam D., Jin Z., Sun X., Xu M. (2024). Predicting gypsum tofu quality from soybean seeds using hyperspectral imaging and machine learning. Food Control.

[5] Colmer J., O’Neill C.M., Wells R., Bostrom A., Reynolds D., Websdale D., Shiralagi G., Lu W., Lou Q., Le Cornu T. (2020). SeedGerm: A cost-effective phenotyping platform for automated seed imaging and machine-learning based phenotypic analysis of crop seed germination. New Phytol..

[6] Sabanci K., Aslan M.F., Ropelewska E., Unlersen M.F. (2022). A convolutional neural network-based comparative study for pepper seed classification: Analysis of selected deep features using a support vector machine. J. Food Process Eng..

[7] Deng X., Liang S. (2023). Detection of rice seed hole-seeding quantity based on deep learning. Proceedings of the 2023 IEEE 6th Information Technology, Networking, Electronic and Automation Control Conference (ITNEC), Chongqing, China, 24–26 February 2023.

[8] Yan Z. (2024). Design and Key Technologies of an Automatic Tomato Plug-Tray Seeding Device and Missed-Seeding Detection System. Master’s Thesis.

[9] Sandhiya M., Visvesh B., Ugendrababu M., Tinisha A. (2024). Varietal seed classification and seed germination prediction system. Proceedings of the 2024 Second International Conference on Emerging Trends in Information Technology Engineering (ICETITE), Vellore, India, 23–24 February 2024.

[10] Bai W., Zhao X., Luo B., Sun H., Wu H. (2023). A YOLOv5-based method for wheat seed germination detection. Acta Agric. Zhejiangensis.

[11] Lang T. (2020). Image-Processing-Based Identification of Foxtail Millet Seed Germination Rate and Measurement of Radicle and Plumule Growth. Master’s Thesis.

[12] Yao Y., Gui Z., Liu H., Yang Z., Yao L., Li K., Lin Z., Mao Y., Jia Z., Li Y. (2026). Tea bud pose estimation and grading detection network based on improved YOLOv7. Front. Plant Sci..

[13] Sun W., Xu M., Xu K., Zhang Y., Zhao L. (2025). CSGD-YOLO: A corn seed germination status detection model based on YOLOv8n. Agronomy.

[14] Shi Q., Zhang Y., Du X., Chen X., Liu G. (2026). Light-YOLO-Pepper: A lightweight model for detecting missing seedlings. Agriculture.

[15] Tasmara J., Supriyanto S., Solahudin M. (2025). Deep learning-based detection of early germination stages in chili pepper (*Capsicum annuum* L.) seedlings under greenhouse conditions. J. Tek. Pertan. Lampung.

[16] Tian D., Wang X., Liu D., Hao Y. (2025). MFDA-YOLO: A multiscale feature fusion and dynamic alignment network for UAV small objects detection. PLoS ONE.

[17] Liu Q., Yu H., Zhang P., Geng T., Yuan X., Ji B., Zhu S., Ma R. (2025). MFEF-YOLO: A Multi-Scale Feature Extraction and Fusion Network for Small Object Detection in Aerial Imagery over Open Water. Remote Sens..

[18] Shi J., Luo W., Wang X., Guo J., Ren H. (2026). FDA-YOLO: A Feature Fusion and Attention-Based Network for Multiscale Tomato Maturity Detection in Real-World Agricultural Scenarios. Sensors.

[19] Deng Y., Hu Y., Ye Y., Xu P. (2026). AD-YOLO: A Unified Method for Traffic-Dense and Small Object Detection in UAV Images. Drones.

[20] Jocher G., Chaurasia A., Qiu J. Ultralytics YOLO, Version 8.3.189. Released 29 August 2025. https://github.com/ultralytics/ultralytics.

[21] Liu W., Lu H., Fu H., Cao Z. (2023). Learning to Upsample by Learning to Sample. Proceedings of the IEEE/CVF International Conference on Computer Vision (ICCV).

[22] Wang Q., Wu B., Zhu P., Li P., Zuo W., Hu Q. (2020). ECA-Net: Efficient Channel Attention for Deep Convolutional Neural Networks. Proceedings of the IEEE/CVF Conference on Computer Vision and Pattern Recognition (CVPR).

[23] Hou Q., Zhou D., Feng J. (2021). Coordinate Attention for Efficient Mobile Network Design. Proceedings of the IEEE/CVF Conference on Computer Vision and Pattern Recognition (CVPR).

